# Iron-rich waste activated sludge-derived vivianite for boosting pak choi growth: fertilizer effectiveness and mechanism elucidation

**DOI:** 10.3389/fpls.2026.1817356

**Published:** 2026-05-26

**Authors:** Xiaodong Xin, Linjuan Li, Boyu Lu, Yilin Niu, Qian Liu, Jiamin Zhang

**Affiliations:** Research Center for Eco-Environmental Engineering, Dongguan University of Technology, Dongguan, China

**Keywords:** fertilizer efficiency, hydroponic cultivation, iron-rich waste activated sludge (WAS)-derived vivianite, microbial mechanism, nutrients elements uptake

## Abstract

This study investigated the fertilizer effectiveness and mechanisms of vivianite, recovered from iron-rich waste activated sludge (WAS) via anaerobic fermentation, for enhancing pak choi growth through hydroponic cultivation. The vivianite-added groups achieved comparable effects to the commercial nutrient solution (NS) group in promoting chlorophyll synthesis, plant height growth, and biomass accumulation and significantly outperformed the control groups. The vivianite could serve as a slow-release fertilizer, continuously supplying nutrients to enhance the growth of pak choi. This enhancement was facilitated by a synergistic collaboration among root-associated microorganisms, including *Burkholderia-Caballeronia-Paraburkholderia, Mucilaginibacter, Caulobacter, Sphingomonas*, and the primary phosphorus-solubilization contributors, namely, *Novosphingobium, Brevundimonas*, and *Pseudomonas.* Moreover, the vivianite improved root-associated microbial diversity and upregulated the activity of a key iron-acquisition protein, the outer membrane receptor. These effects collectively contributed to pak choi growth. Such iron-rich WAS-derived vivianite could represent a promising fertilizer candidate for hydroponic systems, offering a sustainable pathway to repurpose iron-enriched WAS for agricultural use.

## 1 Introduction

Phosphorus (P) is an essential and indispensable element for living organisms, primarily found in the Earth’s crust and within biological systems. It is necessary for human metabolic processes and food supply, playing a crucial role in global biological activity (Jalali and Jalali, 2016). Its main source is non-renewable phosphate rock, whereas the global reserves of sedimentary phosphate deposits are very limited (van Dijk et al., 2016). A large proportion of the P consumed by human activities enters wastewater treatment plants (WWTPs), where it is eventually retained in waste activated sludge (WAS as a P reservoir). Thus, P recovery from WAS is a critical option for obtaining this non-renewable and strategically important resource (Recena et al., 2022). Moreover, inexpensive iron salts are commonly consumed to meet the stringent P discharge standards in current WWTPs in China, for purposes such as chemical P removal (CPR) and improving sludge dewaterability. This extensive use of iron salts enriches the resulting WAS with both iron (Fe) and P. Studies have confirmed that the mineral vivianite [Fe_3_(PO_4_)_2_·8H_2_O] can form in the solid phase of this WAS (Wilfert et al., 2015; Wilfert et al., 2016; Xin et al., 2025), and it can be recovered via magnetic separation (Prot et al., 2019). Such recovered vivianite contributes to plant growth, with positive effects reported for ryegrass (*Lolium multiflorum* L.), tomato (*Solanum lycopersicum* L.) (Sha et al., 2025), bean plants (Fodoué et al., 2015), and the prevention of Fe chlorosis in strawberry plants (de Santiago et al., 2013). However, the large-scale application of vivianite as a fertilizer faces two main obstacles: (i) its high market price (e.g., 10,000 €/ton) (Zhang et al., 2022) and (ii) its limited fertilizer efficiency in soil-based systems due to the complexity of Fe and P transformation in the root microenvironment and vivianite’s inherently low solubility (Ksp 10^-35^-10^-39^ at 25 °C) (Yang et al., 2022). While methods such as citrate exudation can enhance dissolution, they increase application costs (Yang et al., 2022).

Aiming to address these barriers, low-cost iron-rich WAS-derived vivianite has previously been produced successfully (Xin et al., 2025). On the other hand, soilless cultivation systems, such as hydroponics, have increasingly become major cultivation techniques in modern agricultural industries (Savvas and Gruda, 2018). Hydroponics involves growing plants directly in a nutrient solution or an inert medium, replacing soil. This creates a trophic environment that enhances the dissolution efficiency of slow-release fertilizers, allowing for crop production (e.g., vegetables) in areas with limited land availability or unsuitable soil conditions (Gómez et al., 2019). By providing controlled conditions for plant nutrient supply, hydroponics is widely applicable for research, including studies on plant growth, nutrient uptake, and interactions with other available nutrients (Samreen et al., 2017). Consequently, hydroponics can circumvent the soil-related limitations mentioned above and can serve as an ideal testing ground for this low-cost vivianite. However, the fertilizer characteristics of vivianite, including plant growth properties, nutrient uptake, and fertilizer efficiency, as an alternative to commercial hydroponic nutrient solutions for enhancing plant growth through hydroponics have not been clearly investigated. Moreover, the underlying interactive mechanism between vivianite dissolution (for releasing Fe and P) and root microorganisms remains unclear.

In this study, the feasibility of recovered iron-rich WAS-derived vivianite as a slow-release fertilizer was systematically evaluated through hydroponic cultivation of pak choi (*Brassica rapa*). First, the growth characteristics, nutrient absorption, and fertilizer efficiency during the seedling stage in hydroponics were clearly demonstrated. Meanwhile, vivianite dissolution for Fe and P release triggered by the root microbiome was thoroughly explored. Finally, the underlying interactive mechanisms of vivianite and root microorganisms were clearly elucidated. This study aims to develop a low-cost vivianite fertilizer to enhance plant growth, thereby contributing to a circular pathway from waste recovery to agricultural application and food supply.

## 2 Materials and methods

### 2.1 Plant material and growth conditions

The pak choi seeds (belonging to a green-leaf variety of pak choi, *Brassica rapa*) used in this study were purchased from Jinwangjie Seed Co., Ltd. in Xinxiang City, China. The commercial hydroponic nutrient solution was purchased from Zhinong Technology Co., Ltd. in Shangqiu City, China, and used as the control fertilizer. It contained the following essential elements: 58.94 mg/L of N, 12.75 mg/L of P, 93.66 mg/L of K, 36.01 mg/L of Ca, 13.58 mg/L of Mg, 27.82 mg/L of S, 0.27 mg/L of B, 0.01 mg/L of Cu, 1.38 mg/L of Fe, 0.35 mg/L of Mn, and 0.03 mg/L of Zn. The electrical conductivity (EC) of the commercial hydroponic nutrient solution was 760 μS/cm, with a total dissolved solids (TDS) value of 382 ppm and a pH of 5.87. Prior to use, this nutrient solution was diluted 1:500 (v/v) with ultrapure water.

### 2.2 Vivianite preparation and characterization

The vivianite used as fertilizer in this study was produced through biofermentation of iron-rich WAS enhanced by a biomanufacturing hydrolase pretreatment, as mentioned previously (Xin et al., 2025). The detailed hydrolase pretreatment procedure is briefly described in the **Supplementary Materials**. Afterwards, the vivianite formed in the solid phase of the fermented solid residue was concentrated using an electromagnetic separator, accounting for approximately 20%-25% of the total solids in the fermented solid residues (purity, *w*/*w*). The contents of the main nutrient and metallic elements in the recovered fermented solid residues (containing vivianite) were as follows: 3,950 ± 28 mg/kg of N, 5,811 ± 36 mg/kg of P, 811 ± 11 mg/kg of K, 7,798 ± 38 mg/kg of Ca, 357 ± 12 mg/kg of Mg, 8.40 ± 0.52 mg/kg of B, 11,140 ± 45 mg/kg of Fe, and 67.49 ± 2.34 mg/kg of Mn. The contents of heavy metal elements were also determined as follows: 8.29 ± 1.21 mg/kg for As, 0.47 ± 0.03 mg/kg for Cd, 93.45 ± 1.25 mg/kg for Cr, 28.93 ± 1.14 mg/kg for Ni, 34.99 ± 2.35 mg/kg for Pb, 0.26 ± 0.02 mg/kg for Hg, 68.26 ± 2.54 mg/kg for Cu, and 76.96 ± 3.75 mg/kg for Zn. These heavy metal levels were all below the corresponding allowable concentrations regulated by the Chinese National Standards (GB 15618-2018) (Chinese National Standard, 2018), which specify limits of 30 mg/kg for As, 0.6 mg/kg for Cd, 200 mg/kg for Cr, 100 mg/kg for Ni, 120 mg/kg for Pb, 2.4 mg/kg for Hg, 100 mg/kg for Cu, and 250 mg/kg for Zn. Magnetic separation for vivianite enrichment was performed using a magnetic separator, with details described previously (Xin et al., 2025). The recovered fermented sediment was dried in a vacuum freeze-dryer at -70 °C for 48 h. The dried samples were then ground under anaerobic conditions. The phase composition and crystal structure of vivianite in the samples were analyzed using an X-ray diffractometer (XRD, D8 ADVANCE, Bruker, Germany), and the obtained data were processed with JADE 6.5 software. Surface morphology was examined using scanning electron microscopy (SEM, Verios G4 UC, Thermo Fisher Scientific, USA), and surface elements were determined using energy-dispersive spectroscopy (EDS, Oxford X-Max 80, UK). Additionally, high-resolution spectra of Fe 2p and P 2p from the dried samples were analyzed using X-ray photoelectron spectroscopy (XPS, ESCALAB XI, Thermo Fisher Scientific, USA), as described previously (Xin et al., 2025).

### 2.3 Experimental design and treatments

The seedlings used in this study were obtained through seed germination in rectangular boxes (18.0 cm × 12.0 cm × 11.0 cm). Each box was equipped with six planting holes, and soilless carbonized cotton served as the growth medium within these holes. Pak choi seeds were then sown in the carbonized cotton in each hole for germination and maintained at a constant humidity of 90%. The experiment used six groups of 500 mL transparent plastic cups as hydroponic containers. Each group was filled with one of six nutrient substrates: vivianite derived from iron-rich WAS at three addition levels (designated as P-0.5, P-1, and P-2), a diluted commercial nutrient solution (NS), purified water (PW), and local tap water (TW). The P-0.5 group denotes that the P supplied from the added vivianite was equivalent to 50% of the P concentration in the NS group. Likewise, the P-1 and P-2 groups indicate that the P dosage from the added vivianite was, respectively, equal to and twofold higher than the P content in the NS group. The P contents in the vivianite sample and NS were determined using inductively coupled plasma mass spectrometry (ICP-MS, SUPEC 7000, EXPEC Technology, China). The PW and TW groups served as two control groups. Each container was filled with 300 mL of the different cultivation solutions for seedling growth (grown in carbonized cotton), and all groups were conducted with three replicates. The detailed substrate compositions are listed in **Supplementary Table 1** in the **Supplementary Materials**. The hydroponic temperature was controlled at 24 °C with an air humidity of 64%. The light intensity was maintained at 2000 lx, with a photoperiod cycle of 12 h/12 h per day. Batch cultivation was conducted on a 7-day cycle. After six cultivation cycles (i.e., 42 days), approximately five pak choi seedlings were grown in each container, and uniformly grown pak choi seedlings were harvested for chemical analysis. At the beginning and end of each cultivation cycle, hydroponic solution and plant samples were collected regularly for analysis, including measurements of EC, TDS, pak choi leaf count, plant height, and nutrient changes in the hydroponic solution. Moreover, chlorophyll content, plant fresh weight, and dry weight were determined. Pak choi plant tissues were collected for macronutrient, micronutrient, and heavy metal content analyses. Furthermore, plant root microbial samples were collected for analysis at the end of each cultivation cycle, while microbial samples from the carbonized cotton were used as controls.

### 2.4 Sampling and measurement protocols

#### 2.4.1 Plant growth parameters

Plant height was measured using a ruler, while leaf count was determined by direct visual enumeration. The contents of chloroplastic pigments were determined by ethanol spectrophotometry, with measurements at 665 nm, 649 nm, and 470 nm for chlorophyll a, chlorophyll b, and total carotenoids, respectively (Xie et al., 2021). For fresh weight measurement, the plants were washed, dried, and weighed separately. For dry weight measurement, all fresh plants were vacuum freeze-dried at -70 °C before weighing. All weighing procedures were conducted using an analytical balance (METTLER TOLEDO, Switzerland). The chemicals used in this study were of analytical grade.

#### 2.4.2 Chemical analysis of hydroponic solutions

The EC and TDS of various nutrient solutions were measured using a water quality tester (TDS & EC meter, Germany). The net changes in EC and TDS were calculated as the values measured on day 7 of each cultivation cycle minus the corresponding values detected on day 1.

#### 2.4.3 Plant tissue elemental analysis

The elemental contents in plants and hydroponic solutions were determined using inductively coupled plasma mass spectrometry (ICP-MS, SUPEC 7000, EXPEC Technology, China). The pH was measured using a PHS-3E pH meter (INESA, China). A total organic nitrogen analyzer (elementar vario TOC, Germany) was used to determine total nitrogen (TN) content.

#### 2.4.4 Microbial community analysis

Root microbial analysis was performed through high-throughput sequencing using Illumina MiSeq sequencing analysis (Sangon, China). Species taxonomy was identified using the RDP classifier (http://rdp.cme.msu.edu/misc/resources.jsp), while α-diversity estimators of root microbial communities were evaluated by Mothur (version 1.43.0) (http://www.mothur.org/wiki/Schloss_SOP#Alpha_diversity), including operational taxonomic unit (OTU) number, Ace index, Chao index, Shannon index, Shannoneven, and coverage. Functional gene prediction for root microbial communities based on 16S rRNA gene sequences was conducted using the PICRUSt program (version 1.1.4), while the relative abundance of functional genes was predicted according to the Kyoto Encyclopedia of Genes and Genomes (KEGG) database (Qian et al., 2024). The raw sequencing data used in this study have been submitted to NCBI (https://www.ncbi.nlm.nih.gov/) under accession numbers SAMN53688110 to SAMN53688116. The raw data are available upon request.

### 2.5 Statistical analysis

Statistical analysis of the experimental data was conducted using Origin 2018 and SPSS 20.0 software. The data were expressed as mean values ± standard deviation using the Origin 2018 software. One-way analysis of variance (ANOVA) was used to evaluate the significant differences in plant growth parameters, nutrient element changes, relative abundances, and OTU counts of the main root microorganisms among all groups, with a confidence interval of 95% using SPSS 20.0 software. Statistical significance was set at *p* < 0.05. The Z-scores used to establish the heatmap for metabolic function prediction of key enzymes in the root microbial community were calculated according to the formula Z= (X-μ)/σ, as described in a previous study (Peng et al., 2026). Here, Z represents the Z-score, X represents the raw data, μ represents the mean value, and σ represents the standard deviation. A positive Z-score indicates a value above the mean, a negative Z-score indicates a value below the mean, and Z-score of zero indicates a value equal to the mean.

## 3 Results

### 3.1 Characterization of the iron-rich WAS-derived vivianite

**Figure 1:**
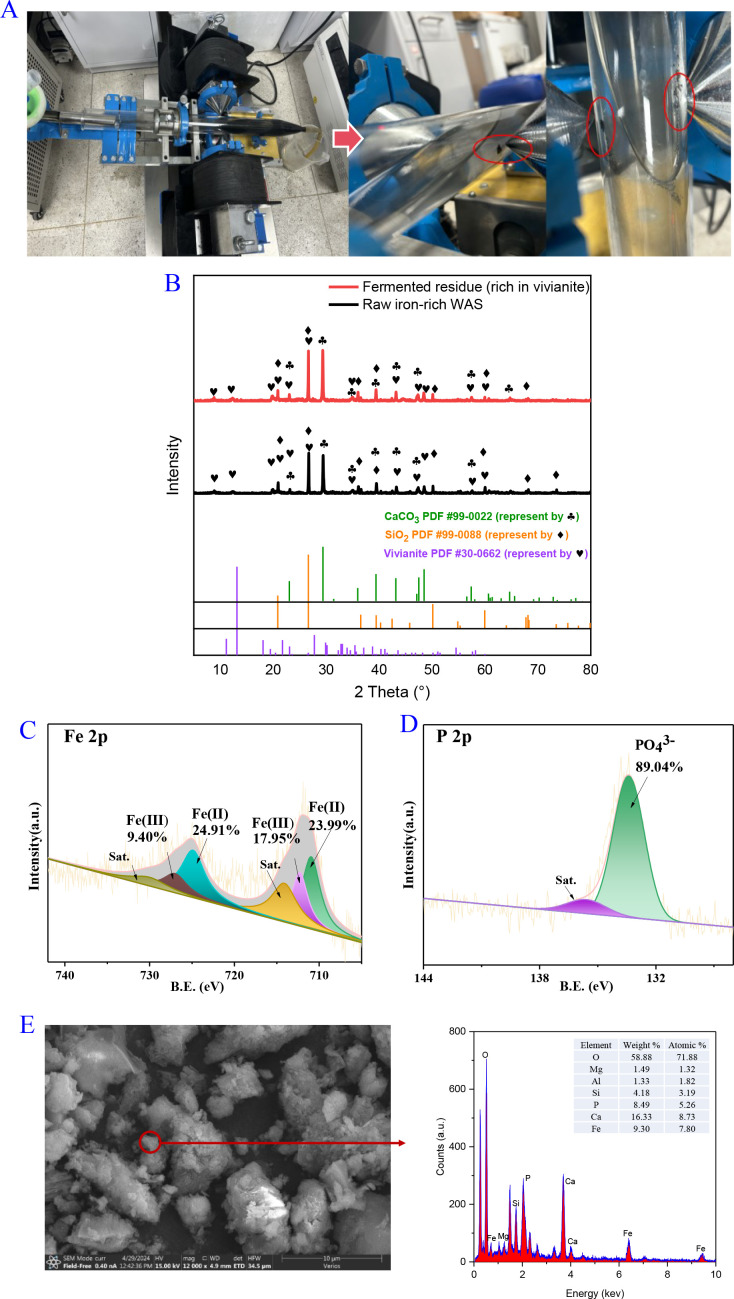
Characterization of iron-rich WAS-derived vivianite from fermented residues, as reported previously (Xin et al., 2025). **(A)** XRD results. **(B)** XPS results of Fe 2p. **(C)** XPS results of P 2p. **(D)** SEM-EDS result. **(E)** Vivianite separation using an electromagnetic separator.

The vivianite, as the slow-release Fe-P fertilizer used in this study, was recovered from fermented iron-rich WAS residues via magnetic separation, as shown in **Figure 1A** and described previously (Xin et al., 2025). The vivianite formed in the magnetically separated iron-rich WAS residues after acidogenic fermentation was characterized and confirmed by XRD, XPS, and SEM-EDS analyses, as shown in **Figures 1B–E**. The XRD results present a series of characteristic peaks at diffraction angles of 13.18°, 18.13°, 23.08°, 26.65°, and 29.49° (**Figure 1B**). Notably, the diffraction angles of 13.18°, 18.13°, and 23.08° correspond clearly to the standard pattern of vivianite (PDF #30-0662) (Wu et al., 2023). Meanwhile, the diffraction angles of 26.65° and 29.49° closely match the standard patterns of SiO_2_ (PDF #99-0088) and CaCO_3_ (PDF #99-0022), respectively. These results indicate the coexistence of vivianite crystals, SiO_2_, and CaCO_3_ in the concentrated vivianite samples. The presence of SiO_2_ is attributed to fine sand present in the waste sludge (Guo et al., 2023), while the formation of CaCO_3_ was triggered by the use of CaO to improve the dewatering properties of iron-rich WAS prior to anaerobic fermentation, as confirmed previously (Xin et al., 2025). More specifically, the intensity of characteristic diffraction peaks of a crystalline phase generally increases with its mass fraction (wt%) in multiphase mixtures according to the semiquantitative principles of XRD. Herein, the variation in diffraction intensity at the characteristic angles of vivianite indicates clear crystalline vivianite formation compared with raw iron-rich WAS (**Figure 1B**). Moreover, XPS was further employed to examine the atomic composition and chemical states of iron and P in the recovered vivianite (**Figures 1C, D**). In the XPS spectra, characteristic signal shifts in the Fe 2p and P 2p regions were clearly observed. The binding energy signals at 712.31 eV and 727.12 eV correspond to Fe 2p _3/2_ and Fe 2p _1/2_ of Fe(III) (Dai et al., 2020), while the signals at 710.35 eV and 724.78 eV are attributed to Fe 2p _3/2_ and Fe 2p _1/2_ of Fe(II), respectively (Zhou et al., 2022). In addition, the P 2p binding energy signal was clearly detected at 133.74 eV (Zhou et al., 2022). These characteristic binding energy signals of vivianite suggest that vivianite formation was achieved through anaerobic fermentation of iron-rich WAS, consistent with previous studies (Wen et al., 2022; Xin et al., 2025). The reduced Fe(II) generated by iron-reducing bacteria (IRB), such as *Geobacter*, *Ercella*, and *Desulfovibrio*, combined with released PO_4_^3-^, contributed to vivianite crystal formation, as confirmed previously (Wen et al., 2022; Wu et al., 2020; Xin et al., 2025). Furthermore, the SEM-EDS results provided additional evidence of vivianite formation by revealing details of the typical microstructures of vivianite crystals (**Figure 1E**). The monitored particles exhibited slightly wrinkled surfaces accompanied by overall smooth, sheet-like, elongated structures, consistent with the typical morphological characteristics of vivianite (Prot et al., 2020). These particle surfaces contained relatively high contents of Fe, P, and O, with an Fe/P ratio of 1.9, which falls within the characteristic range for vivianite (Prot et al., 2019).

### 3.2 Vivianite fertilizer effectiveness evaluation

#### 3.2.1 Seedling growth

**Figure 2:**
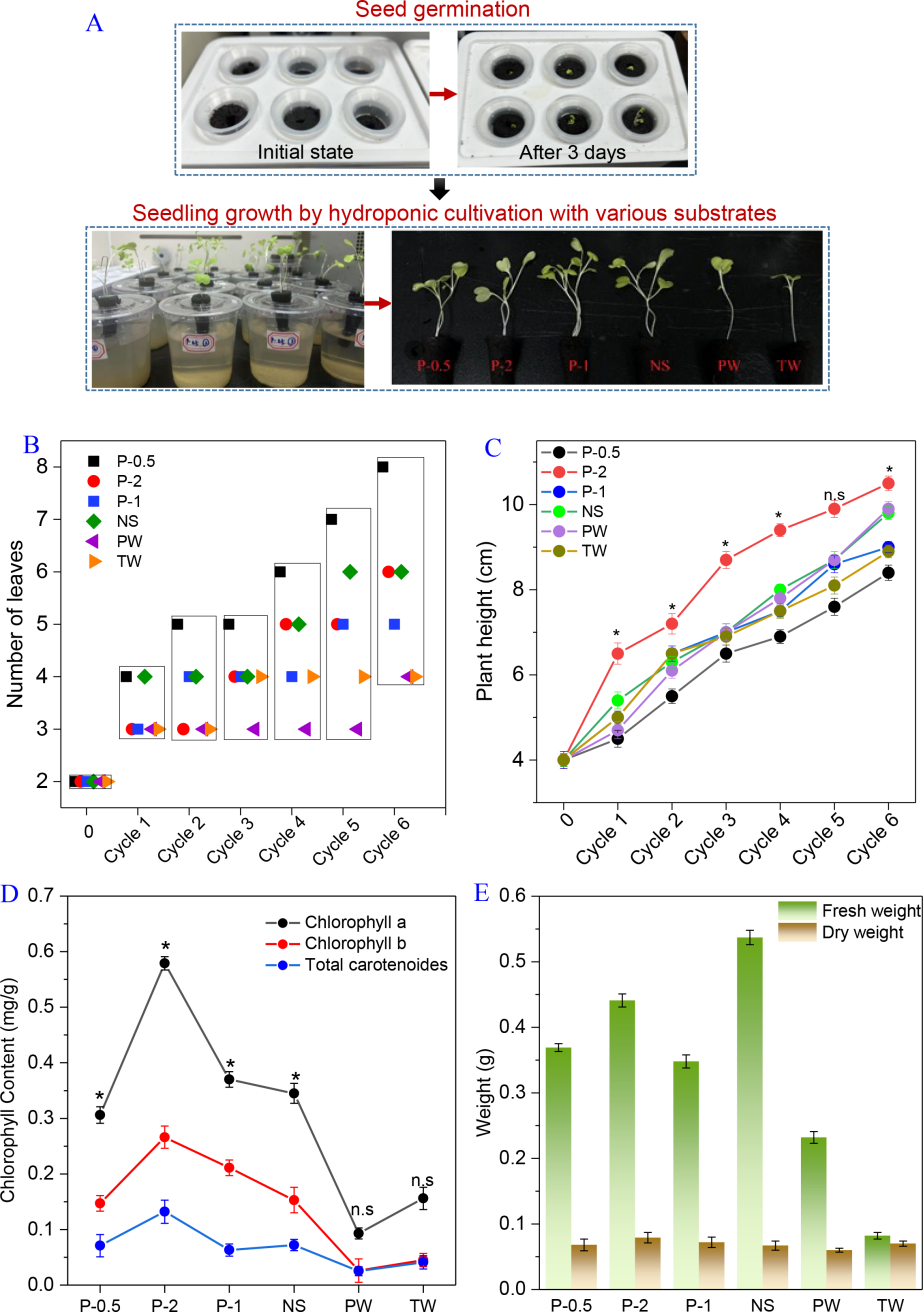
Fertilizer effectiveness evaluation for boosting pak choi seedling growth in all hydroponic groups during different cultivation cycles. **(A)** Seed germination and seedling cultivation under hydroponic conditions in different groups. **(B)** Changes in leaf number. **(C)** Changes in stem height, *p* < 0.05. **(D)** Changes in chlorophyll content, *p < 0.05. **(E)** Changes in fresh and dry weights. NS represents the cultivation group using a commercial nutrient solution as the cultivation substrate. P-0.5, P-1, and P-2 represent groups using recovered vivianite as the substrate at doses of 1.097, 2.194, and 4.388 g/L in purified water, respectively. PW and TW represent the groups using purified water and local tap water, respectively.

The hydroponic cultivation process and morphological parameters of pak choi seedlings under different hydroponic conditions are clearly revealed, including leaf count, plant height, chlorophyll content, fresh weight, and dry weight (**Figure 2**). The seed germination period lasted approximately 3 days. Hydroponic cultivation was then initiated using the obtained seedlings with different nutrient substrates, and the growth morphology of pak choi seedlings was monitored (**Figure 2A**). Changes in leaf count across different cultivation cycles showed that the NS, P-0.5, and P-2 groups exhibited higher values than the P-1, PW, and TW groups (**Figure 2B**). Moreover, regarding the plant height changes (**Figure 2C**), pak choi plants in the P-2 group achieved the greatest plant height compared with the other hydroponic groups. The NS and P-1 groups also exhibited greater plant height levels than the PW and TW groups. Notably, the P-0.5 group displayed the lowest plant height, likely due to greater nutrient allocation toward leaf growth. These results indicate that nutrients released from vivianite contributed to vertical plant growth and promoted leaf development by functioning as an effective fertilizer, consistent with previous findings (Jowett et al., 2018). In addition, changes in chloroplastic pigments, including chlorophyll a, chlorophyll b, and total carotenoids, in the different hydroponic groups are shown in **Figure 2D**. The chloroplastic pigment contents in the P-2 group (0.58 ± 0.01 mg/g for chlorophyll a, 0.27 ± 0.01 mg/g for chlorophyll b, and 0.13 ± 0.02 mg/g for total carotenoids) were optimal compared with those in the P-0.5 group (0.31 ± 0.02 mg/g for chlorophyll a, 0.15 ± 0.01 mg/g for chlorophyll b, and 0.07 ± 0.02 mg/g for total carotenoids), P-1 group (0.37 ± 0.01 mg/g for chlorophyll a, 0.21 ± 0.01 mg/g for chlorophyll b, and 0.06 ± 0.01 mg/g for total carotenoids), NS group (0.35 ± 0.02 mg/g for chlorophyll a, 0.15 ± 0.02 mg/g for chlorophyll b, and 0.07 ± 0.01 mg/g for total carotenoids), PW group (0.09 ± 0.01 mg/g for chlorophyll a, 0.03 ± 0.01 mg/g for chlorophyll b, and 0.03 ± 0.01 mg/g for total carotenoids), and TW group (0.16 ± 0.02 mg/g for chlorophyll a, 0.05 ± 0.01 mg/g for chlorophyll b, and 0.04 ± 0.01 mg/g for total carotenoids). Furthermore, significant differences in fresh weight were observed among the different hydroponic groups for pak choi seedlings. The chloroplastic pigment contents in the P-1 and P-0.5 groups were nearly equivalent to those in the NS group. Meanwhile, the plant fresh weights used to evaluate biomass quality in the vivianite-added groups (P-0.5, P-1, and P-2 groups) were substantially higher than those in the PW and TW groups and only slightly lower than that in the NS group (**Figure 2E**). Comparatively, the dry weight differences among the hydroponic groups were less pronounced, with the P-2 hydroponic system showing the highest plant dry weight (0.08 ± 0.01 g). These results indicate that vivianite effectively promoted chlorophyll biosynthesis in pak choi leaves, thereby potentially enhancing photosynthetic efficiency and promoting plant growth under hydroponic conditions at levels comparable to those of the NS group.

#### 3.2.2 Nutrient uptake characteristics of vivianite by seedlings

**Figure 3:**
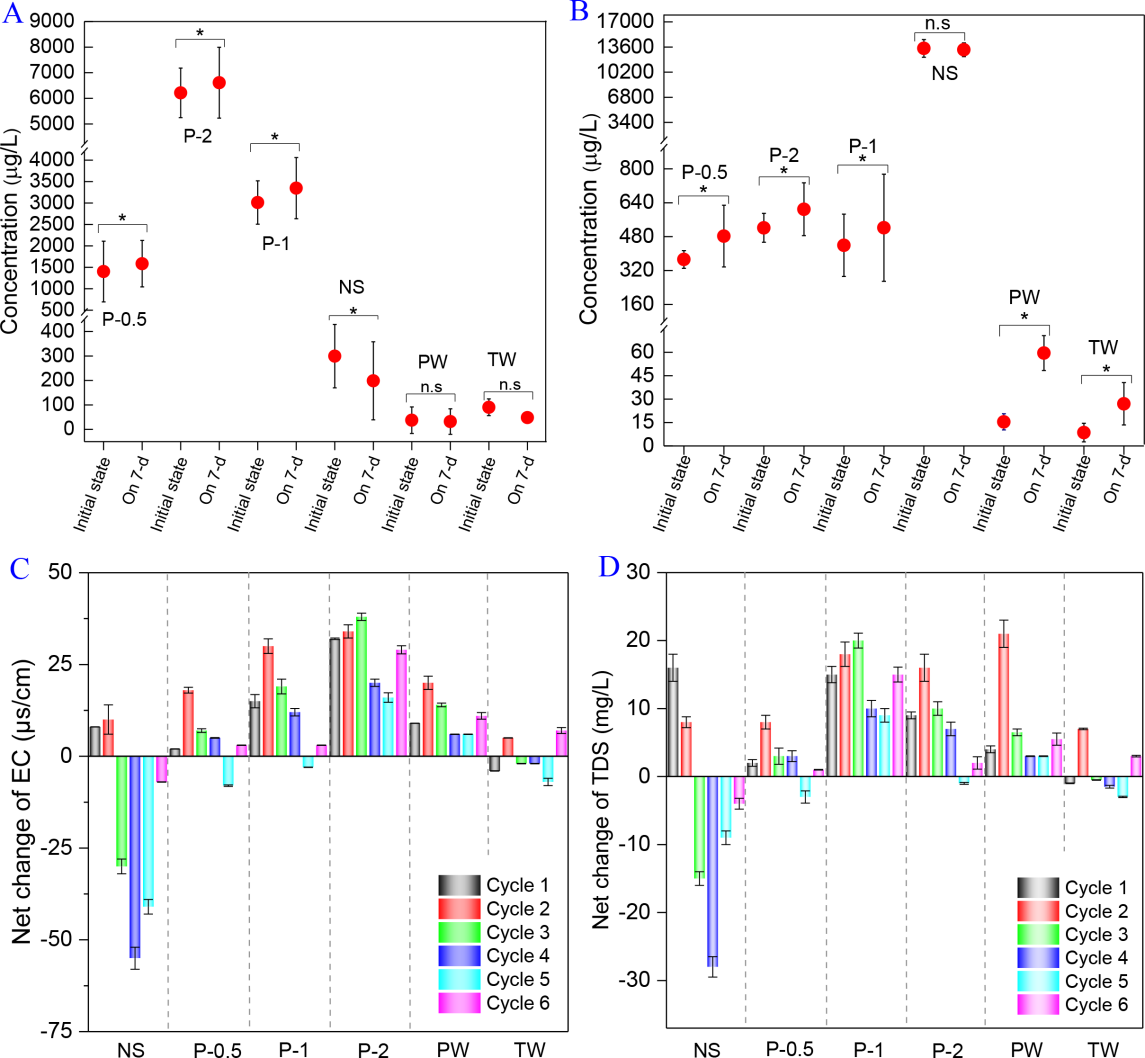
Fe and P concentrations and ionic changes in the hydroponic solutions during cultivation cycles. **(A)** Fe concentration fluctuations. **(B)** P concentration fluctuations. **(C)** EC changes. **(D)** TDS changes. NS represents the cultivation group using a commercial nutrient solution as the cultivation substrate. P-0.5, P-1, and P-2 represent groups using recovered vivianite as the substrate at doses of 1.097, 2.194, and 4.388 g/L in purified water, respectively. PW and TW represent the groups using purified water and local tap water, respectively.

In the liquid phase of various hydroponic solutions, detailed chemical composition strongly influences plant nutrient uptake and root microbial metabolic activities, thereby affecting plant growth and resource utilization efficiency (Cifuentes-Torres et al., 2021; Vandam et al., 2017). To assess nutrient absorption efficiency by seedlings during the hydroponic cultivation process, fluctuations in the macronutrient contents of N, K, Ca, and Mg and the micronutrient contents of B, Cu, Mn, and Zn were analyzed in **Table 1**, while dynamic changes in Fe and P contents are shown in **Figures 3A, B**. During the first, third, and sixth hydroponic cultivation cycles, all cultivation groups exhibited efficient nitrogen uptake (**Table 1**), characterized by a rapid decrease in nitrogen concentration during each respective cycle. Among these elements, N is crucial for plant growth and productivity, serving as a fundamental component of nucleotides, proteins, vitamins, and hormones (Xu et al., 2012). More importantly, the concentrations of other nutrient elements, such as K, Ca, Mg, B, Cu, Mn, and Zn, in the vivianite-added groups (P-0.5, P-1, and P-2) exhibited varying degrees of increase, primarily attributable to the dissolution efficiency of vivianite in the hydroponic solutions exceeding the plant uptake efficiency of these trace elements. This result further indicates that vivianite can be used as a fertilizer supplement with multiple elements. Regarding Fe content changes in **Figure 3A**, the vivianite-added groups (P-0.5, P-1, and P-2) displayed relatively high levels (approximately 1500-1700 μg/L in P-0.5, 3000-3500 μg/L in P-1, and 6100-6900 μg/L in P-2) compared with the NS group and the control groups (PW and TW). These elevated concentrations positively contributed to plant growth, consistent with findings that Fe plays a crucial role in the plant photosynthesis process (Yang et al., 2025). This observation aligns well with the higher chlorophyll levels observed in the P-2 group in **Figure 2D**. Meanwhile, P concentrations fluctuated from 400 μg/L to 600 μg/L in the vivianite-added groups (P-0.5, P-1, and P-2), which were lower than those in the NS group (13,000-13,700 μg/L) but significantly higher than those in the control groups (PW and TW). These results suggest effective P uptake by pak choi seedlings, which is associated with plant photosynthesis and energy transfer processes (Sun et al., 2018). These results suggest that fermented iron-rich WAS residues (enriched with vivianite) used for hydroponic plant cultivation can effectively alleviate nutrient deficiencies and promote plant growth through the simultaneous supplementation of nutrients and trace elements.

**Table 1:** Nutrient contents and main heavy metal changes in the hydroponic solution during cultivation cycles.

Cultivation cycle	Group	Nutrient element content (μg/L)							
		N	K	Ca	Mg	B	Cu	Mn	Zn
Cycle 1 (initial state)	P-0.5	3,437	766	2,905	354	9	10	11	40
	P-2	7,133	995	5,089	407	9	31	28	58
	P-1	4,526	879	3,474	379	11	19	19	44
	NS	57,260	17,121	5,918	2,392	47	3	60	24
	PW	1,853	103	518	32	1	2	1	29
	TW	2,744	566	1,695	288	9	1	2	34
Cycle 1 (on d 7)	P-0.5	2,843	1,348	2,753	488	11	14	15	50
	P-2	4,724	1,099	5,246	455	11	35	33	62
	P-1	3,338	1,343	3,653	525	11	29	24	48
	NS	55,148	16,222	5,593	2,276	40	8	51	23
	PW	3,140	348	313	40	2	1	1	30
	TW	1,853	1,118	1,541	361	9	7	4	29
Cycle 3 (initial state)	P-0.5	4,196	736	2,598	343	9	8	9	32
	P-2	6,803	1,255	5,884	521	13	39	30	63
	P-1	5,483	1,293	3,731	422	11	21	19	49
	NS	52,442	17,953	6,029	2,428	45	3	60	17
	PW	2,447	14	161	12	0.5	1	1	24
	TW	4,229	658	1,790	321	8	2	2	39
Cycle 3 (on d-7)	P-0.5	3,767	781	2,518	353	10	10	9	30
	P-2	4,295	1,869	6,131	619	14	46	41	60
	P-1	4,724	2,385	4,149	491	12	24	20	43
	NS	51,914	16,257	5,567	2,244	39	4	53	42
	PW	3,899	426	239	33	2	2	1	34
	TW	3,734	1,032	1,449	320	10	2	2	22
Cycle 6 (initial state)	P-0.5	6,011	1,213	3,809	470	12	11	8	31
	P-2	7,001	1,568	6,148	596	12	41	35	56
	P-1	5,615	1,348	4,456	514	13	22	17	39
	NS	64,091	19,386	6,629	2,600	46	5	58	98
	PW	3,833	30	258	17	0.3	1	1	19
	TW	4,394	950	2,257	379	8	2	1	31
Cycle 6 (on d-7)	P-0.5	5,615	1,146	3,349	412	33	12	14	34
	P-2	5,813	1,537	6,085	561	30	40	35	53
	P-1	3,239	1,661	3,758	512	25	21	20	39
	NS	55,709	16,304	5,363	2,247	38	2	55	21
	PW	2,810	148	329	32	0.7	1	1	18
	TW	2,414	784	2,129	338	8	2	2	23

NS represents the cultivation group using a commercial nutrient solution as the cultivation substrate. P-0.5, P-1, and P-2 represent groups using recovered vivianite as the substrate at doses of 1.097, 2.194, and 4.388 g/L in purified water, respectively. PW and TW represent the groups using purified water and local tap water, respectively.

In the leaves of pak choi seedlings after six cultivation cycles, the contents of the macronutrients N, P, K, and B; the metal elements Ca, Mg, Cu, Fe, Mn, and Zn; and the heavy metals As, Pb, and Cd are shown in **Table 2**. Plants in the P-2 group exhibited the highest uptake efficiencies for N and P, reaching 62,010 mg/kg and 12,760 mg/kg, respectively. Notably, the N and P uptake efficiencies in the P-1 and P-0.5 groups were comparable to those in the NS group. In addition, plant uptake levels of other elements, namely, B, Fe, and Mn, remained relatively high in the vivianite-added groups (using vivianite as fertilizer) under hydroponic conditions, which contributed to enhanced photosynthesis and growth metabolism, thereby promoting vegetative growth, as supported by the results shown in **Figures 2B–E**. Furthermore, in terms of food safety, the heavy metal contents of As, Pb, and Cd in the plant leaves were below the limits set by the Chinese National Standard (GB 2762-2025) (Chinese National Standard, 2025), which specifies maximum limits of 0.5 mg/kg for As, 0.3 mg/kg for Pb, and 0.2 mg/kg for Cd. Furthermore, the net changes in EC and TDS further corroborated ionic concentration variations across all hydroponic groups (Wan et al., 2025; Shapley et al., 2010), as shown in **Figures 3C, D**. During each cultivation cycle, the net changes in EC and TDS in the hydroponic solutions in the vivianite-added groups increased markedly compared with those in the other groups (NS, PW, and TW). This phenomenon may be caused by two reasons: i) the effective dissolution efficiency of nutrient elements, including K, Ca, Mg, B, Cu, Mn, and Zn, in the vivianite-added hydroponic solutions (P-0.5, P-1, and P-2), which surpassed plant uptake efficiency, as supported by **Table 1** and previous studies (Wan et al., 2025; Shapley et al., 2010); and ii) partial water loss by transpiration, leading to ionic concentration in the solution (Shapley et al., 2010). In detail, the effective absorption capacity of plants in the vivianite-added hydroponic groups, particularly the P-2 and P-1 groups, was comparable to the nutrient absorption efficiency observed in the NS group. These findings further confirm that vivianite can effectively facilitate nutrient uptake and plant growth under hydroponic conditions.

**Table 2:** Main nutrient and metal element contents in pak choi leaves.

Group	Nutrient element content (mg/kg)												
	N	P	K	Ca	Mg	B	Cu	Fe	Mn	Zn	As (0.5*)	Pb (0.3*)	Cd (0.2*)
P-0.5	40,420	10,730	35,300	36,320	4,230	110	0.7	1,146	109	99	0.303	0.254	0.153
P-2	62,010	12,760	48,440	31,330	3,690	120	0.7	766	167	0.5	0.253	0.184	0.094
P-1	50,330	11,480	47,430	29,530	3,950	160	0.3	772	156	83	0.313	0.174	0.163
NS	45,490	11,330	56,950	23,920	3,970	64	0.4	638	141	16	0.183	0.284	0.024
PW	37,790	9,870	41,480	32,860	4,740	150	0.4	1,086	121	0.5	0.153	0.174	0.073
TW	26,750	9,960	33,420	36,560	5,380	148	0.4	1,432	164	131	0.254	0.163	0.014

* represents the limit values listed in the Chinese National Food Safety Standard: maximum levels of contaminants in foods (GB 2762-2025). NS represents the cultivation group using a commercial nutrient solution as the cultivation substrate. P-0.5, P-1, and P-2 represents groups using recovered vivianite as the substrate at doses of 1.097, 2.194, and 4.388 g/L in purified water, respectively. PW and TW represent the groups using purified water and local tap water, respectively.

### 3.3 Root microbial consortia analysis

In terms of microbial ecology, the diversity indices of root microorganisms of all hydroponic groups and the carbonized cotton sample (without seeding seedlings, used as the control) are presented in **Table 3**. All samples exhibited a high coverage index value of 0.999, indicating good reliability of the microbial sequencing data in reflecting the true community structure. Notably, the indices of OTU number, Shannon, Chao, Ace, and Shannoneven in the vivianite-added groups presented slightly higher levels compared with the NS group and substantially higher levels than those in the PW, TW, and carbonized cotton groups. These results indicate that vivianite addition is capable of exerting equivalent effects on reshaping root microbial α-diversity as the commercial fertilizer by promoting greater diversity, richness, and a more even community distribution. Usually, environmental fluctuations (e.g., vivianite addition in this study) can increase overall α-diversity by creating new niches faster than species become extinct, which is thought to provide greater functional stability (realizing vivianite decomposition in this study) (Nguyen et al., 2021; Yuan et al., 2025). Moreover, improved evenness suggests a more uniform relative abundance across different species, which contributes to more robust functionality (Carballa et al., 2015). This is a key factor explaining the effective promotion of pak choi seedling growth by vivianite addition (in **Figure 2**) from the perspective of root microbial ecology.

**Table 3:** Root microbial α-diversity indices based on 16S rRNA gene sequences in all hydroponic cultivations.

Sample	OTUs	Shannon	Chao	Ace	Shannoneven	Coverage
P-0.5	757	5.074	799.663	824.986	0.765	0.999
P-2	719	4.953	762.066	787.234	0.754	0.999
P-1	690	4.925	749.623	780.233	0.747	0.999
NS	659	4.833	703.522	733.825	0.733	0.999
PW	665	4.905	711.204	734.813	0.731	0.999
TW	640	4.692	696.502	728.515	0.723	0.999
Carbonized cotton	440	3.155	488.614	515.863	0.514	0.999

NS represents the cultivation group using a commercial nutrient solution as the cultivation substrate. P-0.5, P-1, and P-2 represent groups using recovered vivianite as the substrate at doses of 1.097, 2.194, and 4.388 g/L in purified water, respectively. PW and TW represent the groups using purified water and local tap water, respectively.

**Figure 4:**
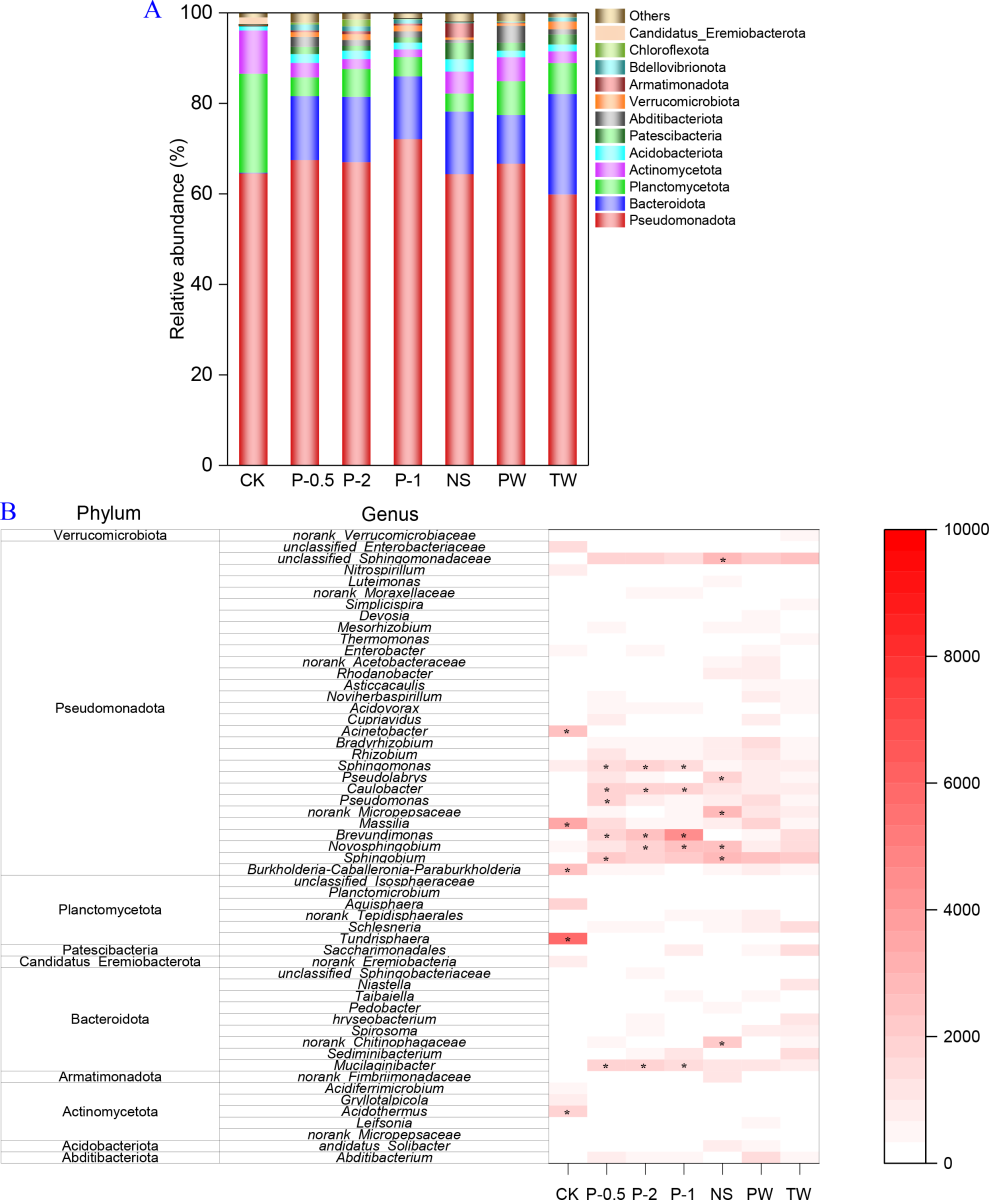
Root microbial community profiles in all hydroponic groups after the sixth cultivation cycle. **(A)** At the phylum level. **(B)** At the genus level based on OTU counts. * represents the *p* < 0.05 as compared with the PW and TW groups. CK represents the microbial sample collected from the carbonized cotton. NS represents the cultivation group using a commercial nutrient solution as the cultivation substrate. P-0.5, P-1, and P-2 represent groups using recovered vivianite as the substrate at doses of 1.097, 2.194, and 4.388 g/L in purified water, respectively. PW and TW represent the groups using purified water and local tap water, respectively.

For the root microbial community profiles at the phylum level (**Figure 4A**), the dominant phyla include *Pseudomonadota*, *Bacteroidota*, *Planctomycetota* [related to nitrogen cycling and degradation of complex organic matter for microbial utilization (Staley et al., 2017)], *Actinomycetota*, and *Acidobacteriota*. Among them, *Pseudomonadota* (relative abundance of 64.34% in the NS group, 66.67% in the PW group, 59.87% in the TW group, 67.46% in the P-0.5 group, 67.00% in the P-1 group, 72.11% in the P-2 group, and 64.53% in the carbonized cotton group) represents one of the most highly diverse and functionally versatile taxa among root microorganisms. It is highly adaptable and plays multiple key roles in hydroponic systems, including the production of plant growth hormones, facilitation of nutrient cycling, and direct promotion of plant growth (Prastowo et al., 2024). *Bacteroidota* (13.85% in the NS group, 10.74% in the PW group, 22.18% in the TW group, 14.14% in the P-0.5 group, 13.86% in the P-1 group, 14.45% in the P-2 group, and 0.15% in the carbonized cotton group), one of the predominant bacterial phyla in hydroponic plant root systems, can promote nutrient release (such as N and P) in hydroponic solutions through organic matter decomposition and participates in carbon cycling to support plant growth (Lage and Bondoso, 2014). Additionally, the relative abundances of *Acidobacteriota* in the P-0.5 and P-2 groups were 1.94% and 1.86%, respectively, higher than those in the PW (1.44%) and TW (1.51%) groups but lower than that in the NS group (2.75%). This taxon is commonly found in soil and rhizosphere environments and contributes positively to organic matter decomposition (Lundberg et al., 2012). As for a key minor member, *Chloroflexota* reached its peak relative abundance (1.48%) in the P-2 group, compared with much lower levels in other groups (e.g., 0.16% in the NS group and 0.18% in the PW group). It plays a significant role in nutrient cycling by participating in carbon and nitrogen transformations within the hydroponic solution, thereby promoting plant photosynthesis and demonstrating strong adaptation to the root environment (Trivedi et al., 2020). In addition, *Acidobacteriota* can adapt to acidic growth conditions and produce active metabolites (Lundberg et al., 2012). Furthermore, *Actinomycetota* promote plant hormone secretion to support growth and development (Zhao et al., 2025), while *Patescibacteria* correlate positively with environmental nitrogen levels and contribute to nitrogen cycling and transformation in hydroponic systems (Lee et al., 2021). These results suggest that vivianite addition shapes the microbial community not only by enriching decomposition-relevant taxa but also by providing bioavailable ionic components that support their metabolic activity and growth.

At the genus level (**Figure 4B**), the detected genera include the predominant taxa *Burkholderia*-*Caballeronia*-*Paraburkholderia*, *Novosphingobium*, and *Brevundimonas*, and the minor taxa *Mucilaginibacter*, *Pseudomonas*, *Caulobacter*, and *Sphingomonas*. Among these, the *Burkholderia*-*Caballeronia*-*Paraburkholderia* group can promote plant nutrient absorption by converting atmospheric nitrogen into plant-available nitrogen fertilizers through nitrogen fixation (Shen et al., 2023). Notably, a peak relative abundance of 40.3% for *Burkholderia*-*Caballeronia*-*Paraburkholderia* was observed in the carbonized cotton group (significantly higher than in the other groups, *p* < 0.05), demonstrating that carbonized cotton is suitable for promoting microbial enrichment for organic matter decomposition. Higher relative abundance levels of *Novosphingobium* were observed in the vivianite-added groups, with significantly higher abundances in the P-1 and P-2 groups than in the other groups (*p* < 0.05). This microbe is a prevalent and dominant genus in plant roots and possesses capabilities for N fixation and P solubilization, enabling it to solubilize P complexes into bioavailable forms through acidification processes (Fan et al., 2022; Wang et al., 2023). P solubilization refers to the process by which microorganisms dissolve insoluble P in the soil by secreting metabolic substances, thereby converting it into available P for plant uptake. This phenomenon may stem from vivianite serving as a P source that promotes the functional enrichment of microorganisms, thereby facilitating P release and plant utilization. Moreover, *Brevundimonas* (significantly more abundant in the P-0.5, P-1, and P-2 groups than in the other groups, *p* < 0.05) possesses the capability to mitigate environmental stress (Singh et al., 2016). The genus *Mucilaginibacter* (significantly more abundant in the P-0.5, P-1, and P-2 groups than in the other groups, *p* < 0.05) is known to possess multiple plant growth-promoting functions, such as production of plant hormones, P solubilization, and N fixation (Lyu et al., 2022). Additionally, *Pseudomonas* in hydroponic plant roots can synthesize plant growth hormones (Prastowo et al., 2024). The *Caulobacter* species (significantly more abundant in the P-0.5, P-1, and P-2 groups than in the other groups, *p* < 0.05) can attach to plant roots and maintain symbiotic interactions with plants (Berrios, 2022). *Sphingomonas* (significantly more abundant in the P-0.5 and NS groups than in the other groups, *p* < 0.05) is renowned for its ability to produce the plant hormone indol-3-acetic acid (IAA) (Pii et al., 2016). These genera exhibited higher relative abundances in the vivianite-supplemented hydroponic groups, suggesting that recovered vivianite contributes to hydroponic pak choi growth as a fertilizer by supplying nutrient elements and facilitating the activity of these decomposers.

**Figure 5:**
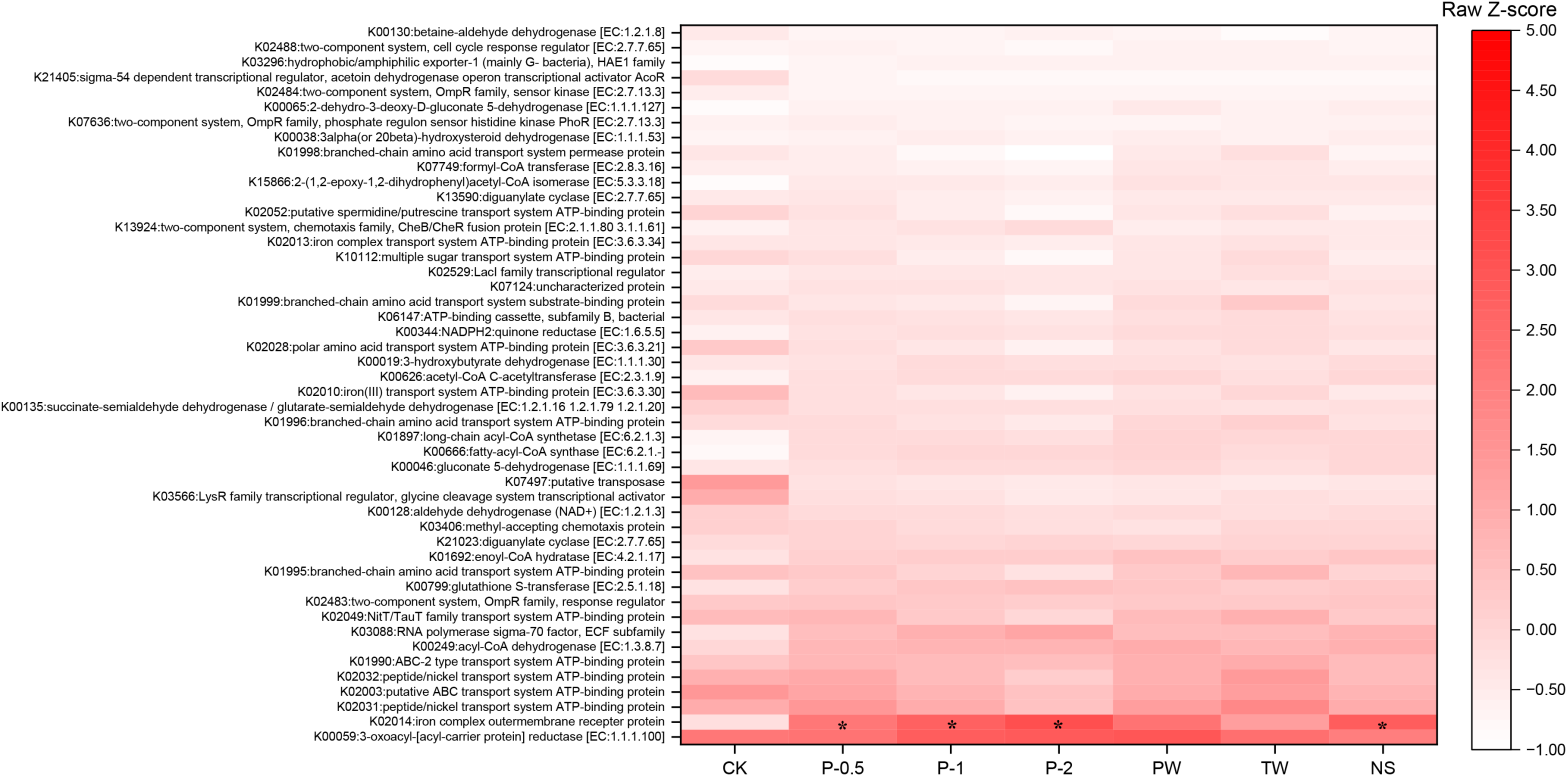
Heatmap of predicted metabolic functions of key enzymes in root microbial communities across all hydroponic groups. Note: CK represents the microbial sample collected from the carbonized cotton. * represents the *p* < 0.05 as compared with the PW and TW groups. CK represents the microbial sample from the carbonized cotton. NS represents the cultivation group using a commercial nutrient solution as the cultivation substrate. P-0.5, P-1, and P-2 represent groups using recovered vivianite as the substrate at doses of 1.097, 2.194, and 4.388 g/L in purified water, respectively. PW and TW represent the groups using purified water and local tap water, respectively.

The relative abundances of functional genes’ associated metabolic functions predicted via the KEGG database to specifically analyze functional changes in root microbial communities are summarized in **Table 4**. The results show that core metabolic pathways related to carbohydrate metabolism, lipid metabolism, metabolism of cofactors and vitamins, energy metabolism, nucleotide metabolism, and amino acid metabolism presented levels in the vivianite-added groups (P-0.5, P-1, and P-2) comparable to those in the NS group and substantially higher than those in the other groups. This result suggests that vivianite addition might contribute to boosting root microbial metabolic activities under hydroponic conditions and facilitating the utilization of small-molecule C, N, and P for plant growth. Although these results were obtained from functional prediction using PICRUSt, they reflect certain changing trends in the metabolic functions of root-associated microorganisms. Furthermore, this finding is supported by the heatmap results for metabolic function prediction of key enzymes in the root microbial community shown in **Figure 5**. Notably, the key enzyme iron complex outer membrane receptor protein was expressed at levels comparable in the vivianite-added groups (P-0.5, P-1, and P-2) to those in the NS group and significantly higher than those in the remaining groups (*p* < 0.05). This functional protein is an important siderophore-related protein involved in iron ion transport and encoded by the TC.FEV.OM genes (Cho et al., 2025). This finding further indicates that vivianite addition during hydroponic cultivation contributes to iron ion transport and acts as a promoter of pak choi growth, consistent with previous findings (Pahari et al., 2017).

**Table 4:** Relative abundances of root microbial metabolic function expression genes based on the KEGG database in all hydroponic cultivations (%).

Carbonized cotton	P-0.5	P-2	P-1	NS	PW	TW	KEGG: pathways
9.019	9.473	9.548	9.441	9.359	9.226	9.362	Metabolism: Carbohydrate metabolism
3.168	3.758	3.717	3.673	3.581	3.432	3.509	Metabolism: Lipid metabolism
3.169	3.299	3.329	3.437	3.224	3.083	3.094	Metabolism: Metabolism of cofactors and vitamins
3.076	3.282	3.259	3.287	3.047	3.059	3.049	Metabolism: Energy metabolism
8.331	8.512	8.632	8.410	8.205	7.956	8.156	Metabolism: Amino acid metabolism
1.505	1.694	1.686	1.628	1.576	1.577	1.577	Metabolism: Nucleotide metabolism
1.345	1.457	1.509	1.536	1.481	1.457	1.519	Metabolism: Biosynthesis of other secondary metabolites
1.836	1.618	1.581	1.541	1.639	1.581	1.528	Metabolism: Metabolism of terpenoids and polyketides
4.363	4.356	4.297	4.223	4.621	4.388	4.412	Metabolism: Xenobiotics biodegradation and metabolism
1.779	1.921	1.916	1.916	1.859	1.702	1.735	Metabolism: Metabolism of other amino acids
0.637	0.603	0.603	0.602	0.550	0.569	0.585	Metabolism: Glycan biosynthesis and metabolism
0.919	1.021	1.091	1.167	0.997	0.993	1.059	Genetic Information Processing: Translation
0.011	0.014	0.017	0.019	0.014	0.013	0.016	Metabolism: Chemical structure transformation maps
35.595	36.352	36.648	36.976	35.844	35.251	35.806	Metabolism: Global and overview maps
5.859	4.684	4.043	3.477	4.334	5.093	3.982	Environmental Information Processing: Membrane transport
5.082	4.735	4.777	4.932	4.401	4.586	4.597	Environmental Information Processing:Signal transduction
5.206	4.572	4.378	4.213	4.315	4.823	4.253	Cellular Processes: Cellular community - prokaryotes
1.256	1.049	1.039	1.059	0.857	1.004	0.999	Cellular Processes: Cell motility
0.572	0.716	0.716	0.705	0.756	0.719	0.744	Organismal Systems: Endocrine system
0.773	1.076	1.125	1.163	1.072	1.066	1.088	Cellular Processes: Cell growth and death
0.269	0.332	0.330	0.320	0.341	0.335	0.339	Cellular Processes: Transport and catabolism
0.024	0.031	0.034	0.037	0.032	0.029	0.032	Organismal Systems: Circulatory system
0.128	0.119	0.121	0.122	0.111	0.112	0.119	Organismal Systems: Immune system
0.189	0.272	0.277	0.280	0.277	0.267	0.278	Organismal Systems: Environmental adaptation
0.128	0.137	0.127	0.121	0.127	0.129	0.123	Organismal Systems: Nervous system
0.023	0.016	0.013	0.011	0.016	0.017	0.015	Organismal Systems: Excretory system
0.029	0.046	0.047	0.047	0.052	0.051	0.048	Organismal Systems: Digestive system

NS represents the cultivation group using a commercial nutrient solution as the cultivation substrate. P-0.5, P-1, and P-2 represent groups using recovered vivianite as the substrate at doses of 1.097, 2.194, and 4.388 g/L in purified water, respectively. PW and TW represent the groups using purified water and local tap water, respectively.

## 4 Discussion

**Figure 6:**
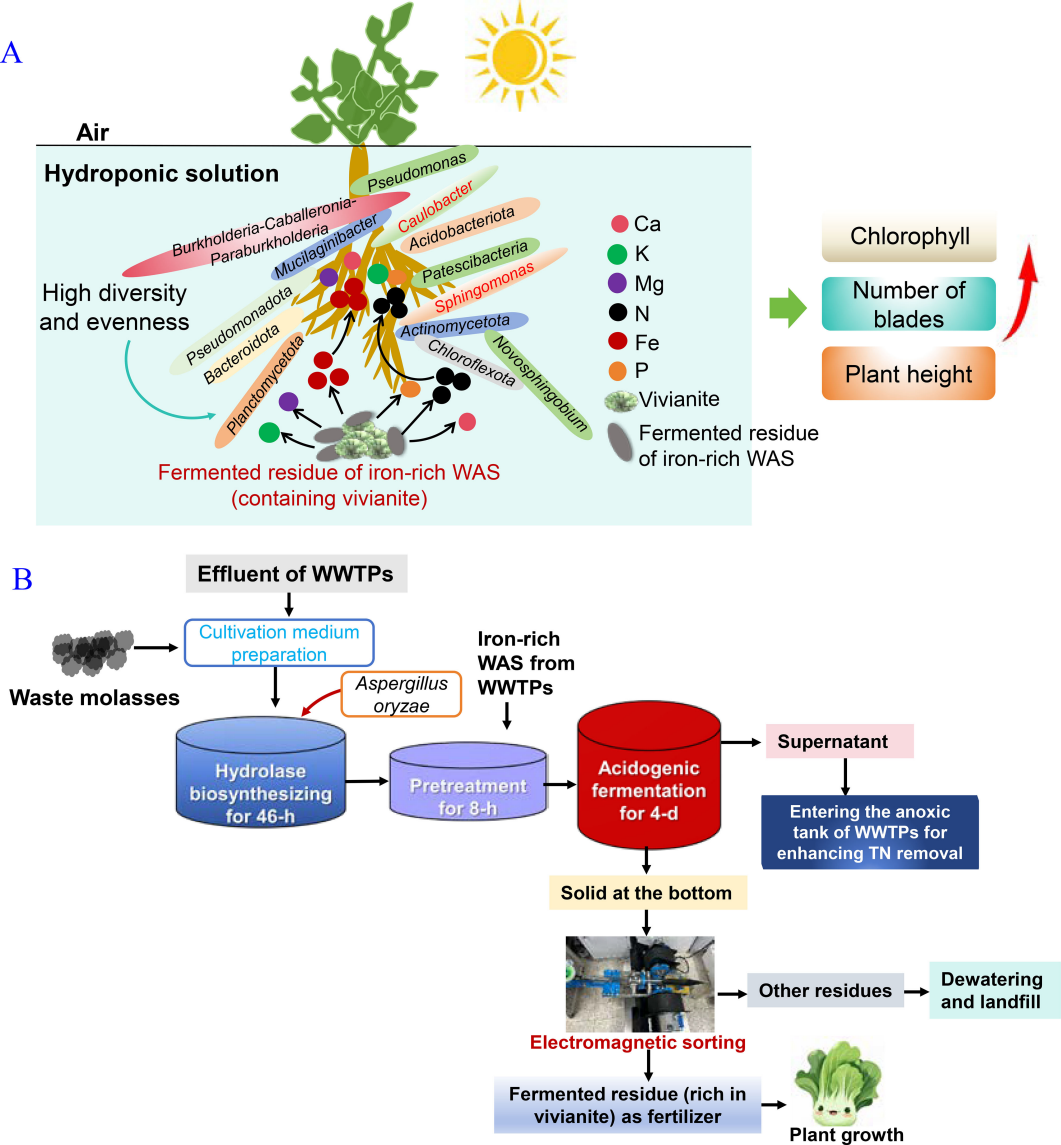
Microbial mechanism and conceptual scheme for iron-rich WAS-derived vivianite obtainment and application. **(A)** Microbial mechanistic elucidation. **(B)** Proposed conceptual application scheme for simultaneous recovery of C, Fe, and P resources from iron-rich WAS and their corresponding applications.

Based on the results shown in **Tables 1**–**4** and **Figures 1**–**5**, a schematic illustration elucidating the microbial mechanism by which vivianite promotes the growth of hydroponic pak choi seedlings is shown in **Figure 6A**. First, the addition of fermented iron-rich WAS residue (containing vivianite) to the hydroponic solution shapes high root microbial diversity and good distribution evenness, which contributes to the formation of a stable and robust microbial community capable of biodecomposing vivianite (Nguyen et al., 2021; Yuan et al., 2025). Subsequently, vivianite dissolution is triggered by core decomposers, including *Burkholderia*-*Caballeronia*-*Paraburkholderia* [which converts atmospheric nitrogen into plant-available nitrogen fertilizers through nitrogen fixation (Shen et al., 2023)], *Novosphingobium* [responsible for N fixation and P solubilization in root communities and capable of solubilizing P complexes into bioavailable forms through acidification processes (Fan et al., 2022; Wang et al., 2023)], *Brevundimonas* [which contributes to phosphate dissolution or secretion of growth-promoting phytohormones (Li et al., 2024)], *Mucilaginibacter* [associated with plant hormone production, P solubilization, and N fixation (Lyu et al., 2022)], *Pseudomonas* [which can synthesize plant growth hormones for plant growth and solubilize P to increase its availability to plants (Prastowo et al., 2024; Ghosh et al., 2015)], *Caulobacter* [which can attach to plant roots and maintain symbiotic interactions with plants (Berrios, 2022)], and *Sphingomonas* [which produces the plant hormone indol-3-acetic acid (IAA) (Pii et al., 2016)]. Overall, the vivianite-added groups were conducive to the enrichment of the major P-solubilizing contributors *Novosphingobium*, *Brevundimonas*, and *Pseudomonas*, thereby facilitating soluble P release from vivianite and boosting plant growth, consistent with previous findings (Fan et al., 2022; Ghosh et al., 2015; Li et al., 2024).

These key members of the root microbial community play critical roles in vivianite dissolution and nutrient release (becoming available substrates for plant uptake) during each cultivation cycle of pak choi. Thus, the fluctuations in macronutrient contents (N, P, K, Ca, Mg, and Fe) observed during hydroponic cultivation are likely attributable to the continuous nutrient release resulting from vivianite dissolution mediated by these core microbes, which supplies essential elements for plant uptake and growth, as supported by the results shown in **Figures 2**, **3** and **Tables 1**, **2**. Through the synergistic cooperation of these root-associated microorganisms, pak choi seedlings grown in vivianite-supplemented hydroponic solutions demonstrated superior performance, including higher chlorophyll content, greater leaf number, and increased plant height. These results suggest that vivianite holds promising potential as an alternative to conventional commercial fertilizers in hydroponic pak choi cultivation. Notably, although the contents of heavy metals, including As, Pb, and Cd, in pak choi leaves were below the maximum contaminant levels established by the Chinese National Food Safety Standard (GB 2762-2025) in this study, the potential risk of heavy metal accumulation exceeding safety standards through long-term use of sludge-derived vivianite may still remain, as mentioned previously (An et al., 2024). Therefore, this sludge-derived vivianite is recommended as an effective fertilizer for cultivating short-duration crops, such as pak choi, lettuce, and green bean, in hydroponic systems.

Based on the above results and the previously reported vivianite preparation from iron-rich WAS fermentation pretreated with biomanufactured hydrolase (Xin et al., 2025), a conceptional application scheme for the simultaneous recovery and utilization of C, Fe, and P sources from iron-rich WAS is proposed in **Figure 6B**. First, hydrolase can be biomanufactured by inoculating hydrolase-producing fungi, *Aspergillus oryzae*, using waste molasses within 46 h. The produced hydrolase (in the cultivation liquid) is then directly applied to pretreat iron-rich WAS (obtained from WWTPs) for 8 h to achieve sludge solubilization. Subsequently, the pretreated iron-rich WAS is fermented for 4 d to simultaneously produce volatile fatty acids (VFAs) and vivianite. The volatile fatty acids (VFAs) in the fermentation liquid phase can enter the WWTPs directly as an external carbon source to enhance TN removal (especially in carbon-deficient WWTPs in southern China), as reported previously (Huang et al., 2020). Meanwhile, the fermented solid residue at the bottom of the anaerobic reactor can be electromagnetically sorted to obtain vivianite, which can then be used as an Fe-P fertilizer to improve plant growth in agricultural systems. Based on this conceptional application scheme, C, Fe, and P sources in iron-rich WAS can be effectively recovered and comprehensively utilized. Additionally, vivianite recovery from iron-rich WAS represents a promising way for converting waste into resource, with positive environmental and economic benefits, according to the economic analysis presented in the **Supplementary Materials**.

## 5 Conclusion

This study systematically evaluated the feasibility and mechanism of using vivianite (recovered from fermented iron-rich WAS residue) as a slow-release fertilizer through hydroponic cultivation to promote pak choi seedling growth. Vivianite showed superior performance in promoting chlorophyll synthesis and plant height and continuously supplied nutrients for pak choi growth through the synergistic cooperation of root-associated microbes, namely, *Burkholderia*-*Caballeronia*-*Paraburkholderia*, *Mucilaginibacter*, *Caulobacter*, and *Sphingomonas*, together with the main P-solubilizing contributors *Novosphingobium*, *Brevundimonas*, and *Pseudomonas*. Such iron-rich WAS-derived vivianite enables a circular pathway that valorizes iron-rich WAS for agricultural application and food production.

## Data Availability

The datasets presented in this study can be found in online repositories. The names of the repository/repositories and accession number(s) can be found in the article/**Supplementary Material**.
