# Supplementary material for: Iron-rich waste activated sludge-derived vivianite for boosting pak choi growth: fertilizer effectiveness and mechanism elucidation
Source: Front Plant Sci. 2026 May 26;17:1817356. doi: 10.3389/fpls.2026.1817356 (PMC13246630; doi:10.3389/fpls.2026.1817356)
Supplement: Supplementary file 1 [file Supplementaryfile1.docx]

**Iron-rich waste activated sludge-derived vivianite for boosting Chinese pak choi growth: Fertilizer effectiveness and mechanism elucidation**

Xiaodong Xin, Linjuan Li, Boyu Lu, Yilin Niu, Qian Liu, Jiamin Zhang*

Research Center for Eco-Environmental Engineering, Dongguan University of Technology, Dongguan, 523808, PR China.

*Corresponding author:

Jiamin Zhang. E-mail: jiaminzhang@dgut.edu.cn.

Address: No.1, University Rd., Songshan Lake District, Dongguan City, Guangdong Province, PR China.

**1. The hydrolase pretreatment process description**

The hydrolase pretreatment was conducted according to a ratio of 1:5 (*v*/*v*, the prepared hydrolase to iron-rich WAS) for 8-h. Herein, about 75 mL of the prepared hydrolase solution and 375 mL of iron-rich WAS were mixed together for 8-h at room temperature with being stirred at a constant speed of 60 rpm by a blender. The pretreated iron-rich WAS samples were collected regularly at an interval of one-hour for detection.

**2. Economic analysis**

In terms of economic analysis of the commercial fertilizer, the cost of preparing 900 mL of the hydroponic solution used in this study (for three parallel samples) is nearly ¥ 0.09 (yuan, RMB) based on the market price of ¥10.00 for purchasing 100 L of the commercial nutrient solution as liquid fertilizer. As for the vivianite preparation cost (taking P-2 group as an example in this study), the P content from nearly 3.95 g of the vivianite sample is used in P-2 group for preparing 900 mL hydroponic solution (for three parallel samples) in this study according to the P content determination result. In the previous study (Xin et al., 2025), about 29.11g/L of fermented iron-rich WAS residue can be obtained after the fermentation by using 75 mL of hydrolase solution for treating 375 mL of iron-rich WAS (initial TS of 32.40 g/L). According to the vivianite purity of 25% in such fermented residue, about 2.73 g vivianite can be obtained from the fermented iron-rich WAS residue (29.11g/L× 0.375 L×25%=2.73 g vivianite). Thus about 543 mL of iron-rich WAS can produce 3.95 g vivianite, which needs about 109 mL of hydrolase according to a 1:5 adding ratio (*v*/*v*, hydrolase to iron-rich WAS). Such 109 mL of hydrolase may cost ¥ 0.007 based on the hydrolase preparation price of ¥ 64.14 for per metric ton, as reported previously (Xin et al., 2025). Besides, the electricity consumption for electromagnetic sorting is about 0.05 kWh, which is calculated by the electrical power of 250 W multiplied by the sorting time of 0.2-h. Such electricity consumption may cost ¥ 0.04 according to the industrial and commercial electricity price of ¥ 0.80 in China. Thus the total cost for vivianite preparation is about ¥ 0.047, which just accounts for 52.2% of the cost of purchasing commercial fertilizer.

**Reference:**

Xin, X., Li, L., Lu, B., Liu, L., Zhang, L., Yang, Y., Li, W., Liu, Q., He, J., He, G., Lv, S.H., Yan, W.W., Luo, L.W., 2025. A novel hydrolase biomanufacturing-driven strategy for boosting production of volatile fatty acids and vivianite in iron-rich waste activated sludge fermentation. Resour. Environ. Sust. 22, 100264.

**Table S1** The detailed composition of various nutrient substrates.

| Cultivation group | Substrate source |
| --- | --- |
| NS | Commercial nutrient solution |
| P-0.5 | 1.097 g of recovered vivianite for per liter purified water |
| P-1 | 2.194 g of recovered vivianite for per liter purified water |
| P-2 | 4.388 g of recovered vivianite for per liter purified water |
| PW | Purified water |
| TW | Local tap water |

Note: For P-0.5, P-1, and P-2, the respective quantities of recovered vivianite were calculated based on the P content in the nutrient solution (12.75 mg/L) and that in the recovered vivianite (5,811 mg/kg).

**Supplementary information:**

**1. Inductively Coupled Plasma Mass Spectrometry Analysis:**

**(1) Digestion Method (acid type, temperature, time):**

Digestion was performed using an aqua regia system (9 mL concentrated hydrochloric acid and 3 mL concentrated nitric acid, volume ratio 3:1). A graphite digestion instrument was used to heat the mixture to 300 °C, with the endpoint determined by the solution becoming clear, typically requiring approximately 1–2 hours to ensure complete decomposition of the organic matrix and thorough dissolution of the target elements.

**(2) Quality Control Measures (certified reference materials, spiked recovery, blank):**

For each batch, certified reference materials were simultaneously analyzed. The accuracy and matrix compatibility of the method were verified by comparing the measured values with the certified values of the reference materials. Prior to sample digestion, a mixed standard solution of known concentration (spike concentration equivalent to 0.5–2 times the background concentration of the sample) was added. Recovery rates were determined after digestion, with acceptable recovery ranging from 80% to 120%, serving to evaluate the accuracy of both sample pretreatment and instrumental analysis. A reagent blank (containing only aqua regia and ultrapure water, without any sample) was prepared in each batch to monitor reagent contamination, environmental background, and instrument carryover. The blank value was subtracted during analysis to eliminate systematic errors.

**(3) Instrument Detection Limit:**

1 μg/L (1 ppb).

**2. Chlorophyll Extraction Using Ethanol Spectrophotometry:**

**(1) Extraction time and temperature, light protection during extraction, and formula used for calculation:**

Approximately 0.1 g of fresh sample was weighed, cut into small pieces, and placed in a mortar. A small amount of quartz sand, calcium carbonate powder, and 3–5 mL of 95% ethanol were added, and the mixture was ground into a homogenate. Grinding continued until the tissue turned white, followed by static incubation in a 75 °C water bath for 3–5 min. After filtration, the volume was adjusted to 10 mL with ethanol. All the above operations were performed under dark conditions. Using 95% ethanol as a blank, the absorbance was measured at wavelengths of 665 nm, 649 nm, and 470 nm, denoted as A665, A649, and A470, respectively.

Concentration calculation formulas:

Ca = 13.95 × A_665_ – 6.88 × A_649_

Cb = 24.96 × A_649_ – 7.32 × A_665_

Cx = (1000 × A_470_ – 2.05 × Ca – 114.8 × Cb) / 245

Content calculation formula (mg/g):

Chlorophyll content (mg/g) = (Chlorophyll concentration × volume of extract) / fresh weight of the sample.

**Details of Microbial Analysis:**

(2) Hypervariable region of the 16S rRNA gene sequenced:

V3–V4 region

(3) Primers and conditions for PCR amplification:

The V3–V4 region of the 16S rRNA gene was amplified using the primer pair 341F (5′-CCTACGGGNGGCWGCAG-3′) and 805R (5′-GACTACHVGGGTATCTAATCC-3′).

The reaction conditions were as follows: an initial denaturation step at 95 °C for 3 min; followed by five cycles of denaturation at 95 °C for 30 s, annealing at 45 °C for 30 s, and extension at 72 °C for 30 s; this was followed by 20 cycles of denaturation at 95 °C for 30 s, annealing at 45 °C for 30 s, and extension at 72 °C for 30 s; and a final extension step at 72 °C for 5 min. The PCR products were stained with ethidium bromide (EB) following electrophoresis on a 2% agarose gel and visualized under ultraviolet light.

(4) Sequencing platform details (MiSeq, read length):

Paired-end sequencing was performed using the Illumina MiSeq™/HiSeq™ sequencing platform. The sequencing reads contained barcode sequences, as well as primer and adapter sequences introduced during sequencing. The primer and adapter sequences were first removed, and paired-end reads were merged into a single sequence based on their overlap. The samples were then identified and differentiated according to the barcode sequences to obtain data for each sample. Finally, quality control and filtering were applied to the data for each sample to obtain valid sequences for downstream analysis. The read length was 2 × 300 bp.

(5) Bioinformatics pipeline parameters (quality filtering thresholds):

The raw paired-end sequencing data were processed using the Usearch software pipeline.

Quality control parameters were as follows:

Paired-end reads were merged based on their overlapping region, requiring a minimum overlap length of 50 bp and allowing a maximum mismatch rate of 0.1;

The quality filtering threshold was set to Q20 (i.e., base quality score ≥ 20), and sequences with an average quality score below this threshold were removed;

Sequences that were too short (< 400 bp) or contained ambiguous bases (N) were discarded;

Denoising/clustering was performed using the DADA2 method to generate amplicon sequence variants (ASVs) or operational taxonomic units (OTUs, clustered at 97% similarity).

(6) Specification of the DNA extraction kit used, clarification that samples were sequenced in a single run to avoid batch effects for PICRUSt analysis, and mention of the reference database used (e.g., Greengenes 13.5):

Genomic DNA was extracted from the samples using the FAST DNA Kit (MoBio, USA). Accurate quantification of genomic DNA was performed using the Qubit 3.0 DNA Assay Kit (MoBio, USA) prior to PCR amplification.

All samples were sequenced in a single batch run to eliminate potential batch effects arising from inter-batch sequencing variability, thereby ensuring the reliability and comparability of downstream PICRUSt2 functional prediction analyses.

Reference databases: Greengenes 13.5, RDP (Ribosomal Database Project).
